# The Role of Neuropeptide-Stimulated cAMP-EPACs Signalling in Cancer Cells

**DOI:** 10.3390/molecules27010311

**Published:** 2022-01-05

**Authors:** Zhengyin Gao, Weng I Lei, Leo Tsz On Lee

**Affiliations:** 1 Cancer Centre, Faculty of Health Sciences, University of Macau, Taipa, Macau, China; zhengyingao7@163.com (Z.G.); leiwengi1996@gmail.com (W.I.L.); 2 Centre of Reproduction, Development and Aging, Faculty of Health Sciences, University of Macau, Taipa, Macau, China; 3 MOE Frontiers Science Center for Precision Oncology, University of Macau, Taipa, Macau, China

**Keywords:** G protein-coupled receptor, exchange proteins directly activated by cAMP (EPAC), cAMP, cancer

## Abstract

Neuropeptides are autocrine and paracrine signalling factors and mainly bind to G protein-coupled receptors (GPCRs) to trigger intracellular secondary messenger release including adenosine 3′, 5′-cyclic monophosphate (cAMP), thus modulating cancer progress in different kind of tumours. As one of the downstream effectors of cAMP, exchange proteins directly activated by cAMP (EPACs) play dual roles in cancer proliferation and metastasis. More evidence about the relationship between neuropeptides and EPAC pathways have been proposed for their potential role in cancer development; hence, this review focuses on the role of neuropeptide/GPCR system modulation of cAMP/EPACs pathways in cancers. The correlated downstream pathways between neuropeptides and EPACs in cancer cell proliferation, migration, and metastasis is discussed to glimmer the direction of future research.

## 1. Neuropeptides and Their Receptors

Neuropeptides are small chain polypeptides that are synthesized and released by neurons through a regulated secretory route. They are the largest and most diverse group of intercellular signalling molecules and can function as neurotransmitters, neuromodulators, autocrine/paracrine regulators, and hormones in the neuroendocrine and nervous system [1,2,3]. Neuropeptides play a critical role in the regulation of exocrine and endocrine secretion, smooth muscle contraction, pain transmission, fluid homeostasis, hypertension, and inflammation. They also regulate food intake, body temperature, and behavioural responses in the central nervous system [4].

Neuropeptides are classified into different subfamilies based on their functions or the structures of their precursors and mature peptides. In mammals, these neuropeptide precursors are encoded by numerous genes that comprise a superfamily of more than 70 genes belong to 18 classical subfamilies (including, opioids, vasopressin/oxytocin, cholecystokinin (CCK)/gastrin, somatostatin, F- and Y-amides, calcitonin, natriuretic factor, bombesin-like (BB-like) peptide, endothelin, glucagon/secretin, corticotrophin-releasing hormone (CRH) and CRH-related peptides, kinin and tensin, neuromedins, motilin, galanin, gonadotropin-releasing hormone (GnRH), neuropeptide B/W, and insulin/relaxins) [1].

Neuropeptides mainly target and bind to G protein-coupled receptors (GPCRs) and trigger diverse intracellular signalling transduction pathways [5]. GPCRs are the largest family of membrane-bound receptors in mammals. They are structurally characterized by the presence of a seven-transmembrane α-helix bundle (7TM) connected by alternating intracellular and extracellular loop regions [6,7]. This protein family is essentially responsible for regulating most of the physiological and pathological pathways and mediates cellular responses of highly versatile ligands such as lipids, nucleosides and nucleotides, amino acids, biogenic amines, inorganic ions, small molecules, peptides, proteins, and even photons. GPCRs are classified into six sub-classes according to their structural and functional similarities: rhodopsin-like family (class A), secretin receptor family (class B), glutamate family (class C), fungal mating pheromone receptors (class D), cAMP receptors (class E), and frizzled/taste 2 (class F). Of these six classes of GPCRs, class D, E, and F do not exist in humans [7,8,9,10,11]. Neuropeptides in humans act via class A and class B GPCRs that share a common origin with the cAMP receptors (Class E GPCRs) [12,13,14].

Neuropeptides interacting with their corresponding GPCRs can activate the Gα protein including G_q_, G_12_, G_i_, and G_s_, of the heterotrimeric complex. They can induce exchange of GDP bound to Gα for GTP and promote their dissociation with the Gβγ subunits. Neuropeptide-GPCRs interactions can lead to the synthesis of classic second messengers such as intra-cytoplasmic calcium (Ca^2+^), diacylglycerol (DAG), and cAMP. These can stimulate enormous cellular changes such as actin organization or rapid tyrosine phosphorylation of multiple substrates including the non-receptor tyrosine kinases PAK and Pyk2 and the adaptor proteins p130 _CAS_ and paxillin [4].

## 2. cAMP-EPAC Signalling

cAMP is a major secondary messenger in cell signalling that is derived from adenosine triphosphate by adenylate cyclase (AC) and regulates many biological processes. After ligand binding to GPCR, the activated subunits of G proteins interact with the membrane-bound AC to form active dimers that trigger cAMP production. Mammals have five main cAMP effector protein families: cAMP-dependent protein kinase A (PKA) [15], exchange proteins directly activated by cAMP (EPACs) [16,17], cyclic nucleotide regulated ion channels (CNG and HCN) [18], cyclic nucleotide receptor involved in sperm function (CRIS) [19], and Popeye domain containing (POPDC) proteins [20].

**Figure 1:**
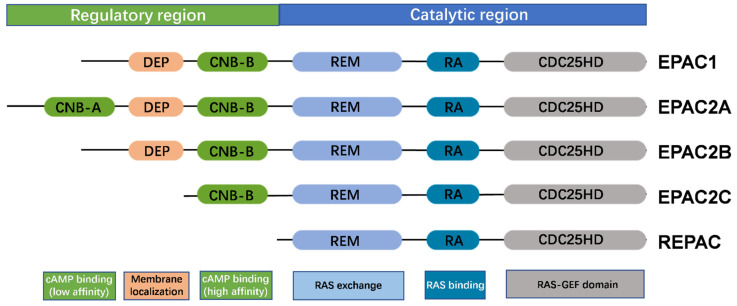
Domain architecture of EPAC1 and EPAC2 isoforms. EPAC1 and EPAC2 isoforms consist of N-terminal cAMP-binding domains and C-terminal guanine nucleotide exchange factor (GEF) domain. The REPAC isoform lacks the cAMP binding (CNB) domain. The N-terminal regulatory region includes a disheveled Egl-10 pleckstrin homology domain and one or two cyclic nucleotide binding domains. The C-terminal catalytic region contains a RAS exchange motif, a RAS-association domain, and a CDC25 homology domain.

EPACs have similar protein structure as other cAMP-activated guanine nucleotide exchange factors (cAMP-GEFs) including N-terminal cAMP-binding domains and a C-terminal GEF domain. Phylogenetic analysis has shown that the N-terminal of EPAC—an autoinhibitory regulation domain probably evolved from the R subunit of PKA—is highly homologous to the Ras-GEF proteins [21]. According to the sequences and domain structure, three different isoforms of EPAC protein family were identified and defined as EPAC1, EPAC2 and REPAC (for related to EPAC). They consist of N-terminal autoinhibitory region (a dishevelled Egl-10, pleckstrin homology domain, and cyclic nucleotide binding domains) and C-terminal catalytic region (a RAS exchange motif, a RAS-association domain, and a CDC25 homology domain) (Figure 1) [22,23,24].

**Figure 2:**
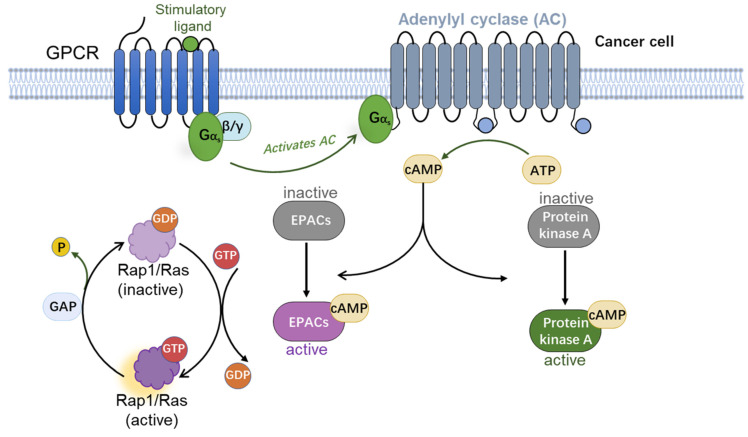
Schematic of cAMP-EPAC/PKA signaling pathways: As soon as the ligands binding to GPCRs, adenylyl cyclase (AC) is activated by Gαs protein-coupled GPCRs and produces numerous cAMP molecules from ATP. cAMP eventually activates protein kinase A (PKA) and EPACs. Activated EPACs function as guanine nucleotide exchange factors (GEFs), leading to a GDP to GTP exchange on both Rap1 and Ras.

The REPAC isoform is functionally deficient because it lacks the cyclic nucleotide binding (CNB) domain [21,25]. The N-terminal of EPAC2 contains two CNB domains: CNB-A and CNB-B. The cAMP binding affinity of CNB-A (Kd value = 87 µM) is lower than the one of CNB-B (Kd value = 1.2 µM), which are determined through isothermal titration calorimetry (ITC). Phylogenetic analysis suggests that EPAC1 evolved from EPAC2 by gene duplication, and lost the CNB-A domain but retained the CNB-B domain [21]. The GEF domain activates the small GTPases by promoting GDP exchange for GTP to trigger downstream Rap1. Ras signalling transduction can then regulate many molecular and cellular events including cell proliferation, cell adhesion, cellular junction interactions, and secretions as well as physiological level biochemical processes such as calcium dynamics, learning and memory, and axonal growth [16,17] (Figure 2). Many studies have also reported the functions of EPACs in human diseases including neurological disorders, cardiovascular diseases, chronic pain, inflammation, kidney diseases, and cancers.

EPAC1 is encoded by the *rap guanine nucleotide exchange factor 3* (*Rapgef3*) gene located on chromosome 12 (12q13.11: 47,734,363–7,771,040) in human genome [26]. *Rapgef3* has 20 splice variants, but only three are validated: transcript variants 1 (6239 nt; encodes EPAC1A (923 aa), transcript variants 2 (5773 nt), and 3 (6003 nt). The latter two encode the same protein EPAC1B (881 aa) [26]. EPAC1 is universally and highly expressed in human tissues including heart, brain, ovary, kidney, blood vessels, adipose tissue, skeletal muscle, central nervous tissue, and uterus [26].

EPAC2 is encoded by *rap guanine nucleotide exchange factor 4* (*Rapgef4*) gene located on chromosome 2 (2q31: 172,735,274–173,052,893) in the human genome [27]. *Rapgef4* has 15 splice variants, and five are validated: EPAC2A is the longest splice variant and consists of 4301nt (1011aa). The other four variants (EPAC2B–EPAC2E) are composed of 3707–4120 nt with 867–791 aa. EPAC2B and EPAC2C lack of CNB-A domain at the NH_2_ terminus. EPAC2 has a tissue specific expression in central nervous system, heart, kidney, pancreas, pituitary, adrenal gland, and liver [28,29]. Interestingly, the expression of different EPAC2 variants vary with tissues: EPAC2A is mainly expressed in central nervous system, pancreas, and pituitary [28]; EPAC2B is expressed in the adrenal glands [30]; and EPAC2C is liver-specific [31].

Several chemically modified cAMP analogs were used as EPAC-selective pharmacological modulators for therapeutic purpose and elaborating the functions of EPAC in physiology and pathophysiology [32,33]. These chemically modified cAMP analogs can be classified into three groups according to their target proteins: EPAC1 only, EPAC2 only, and both EPAC1 and EPAC2. 8-(4-chloro-phenylthio)-2′-O-methyladenosine-3′,5′-cyclic monophosphate (also named 007 or 8-pCPT-2′-O-Me-cAMP) is identified to be a specific and efficient activator for EPAC1. It is about 100-fold sensitive to EPAC1 over PKA due to lack of 2′-hydroxyl group, which is necessary for binding to PKA [32,34,35], and is barely activates EPAC2 [36]. CE3F4, a EPAC1 antagonist, doesn’t compete with cAMP and its antagonistic potency raises at higher level of cAMP concentration [34]. Sp-8-BnT-cAMPS (or S-220) activates EPAC2 efficiently, and barely activates EPAC1 and PKA [36,37]. Both EPAC specific inhibitor 05 (ESI05) and 07 (ESI07) are EPAC2-specific antagonists and are identified by fluorescence-based high-throughput screening among 14400 small molecules [38,39]. These two antagonists bind allosterically to the interface formed by the CNB-A and CNB-B domains of EPAC2 [39]. ESI-08 and ESI-09 inhibit both EPAC1 and EPAC2 though binding to the EPAC1 and EPAC2-conserved CNB-B domain [40,41]. The discovery of EPACs agonists and antagonists helps exploring the functions of EPACs in multiple biological processes. Furthermore, EPAC proteins targeted drugs could also be the candidates for therapy towards cAMP pathway-mediated diseases in the future.

## 3. Neuropeptide and EPAC Signalling Pathways in Cancer

Despite the well-known neuronal and neuroendocrine functions, neuropeptides are potent regulators of cancer progression and metastasis. Neuropeptides are crucial factors in both stimulation and suppression of tumour cell growth via autocrine and paracrine signalling [3,4,42,43]. Studies have also demonstrated that the nervous system promotes tumour metastasis by modulating metastatic cascades through the release of neurotrophins, neurotransmitters, and neuropeptides from nerve fibres surrounding and embedded in the tumour [44].

The function of EPAC1 in cancer progress is well studied, but research about EPAC2 in cancer progression is relatively limited. It is generally believed that EPAC1 has dual functions on cancer proliferation and metastasis. As shown in Table 1, EPAC1 plays a pro-proliferative role in many cancers including lung cancer [45,46,47,48,49], melanoma [50,51,52,53,54], pancreatic cancer [41,55,56,57], cervical cancer [58], fibrosarcoma [59], gastric cancer [60], and rectal cancer [61]. However, several research reported that EPAC1 depict a protective role in glioblastoma [62,63,64], bladder cancer [65] and renal cancer [66]. What’s more, EPAC1 can either promote or attenuate cancer progression in breast cancer [67,68,69], leukaemia [70,71], neuroendocrine cancer [72], ovarian cancer [73,74,75,76] and prostate cancer [77,78,79,80]. The reason for dual functions of EPAC1 in the same cancer is very complicated and can be influenced by cancer cell types as well as genomic and transcriptomic differences, but the integrated mechanism remains elusive. Indeed, the function of EPAC2 in cancer and related regulation pathways still requires further study. Recent research suggests that EPAC2 induces cell apoptosis by mediating the expression of histone deacetylases 8 (HDAC8) in H1299 non-small cell lung cancer cells [81]. The activation of EPAC2 suppresses HDAC8 protein degradation inducing apoptosis through the PI-3-kinase (PI3K)/ Protein kinase B (AKT)/MKK4/JNK1 pathway in lung cancer cells [81]. The section below describes the role of neuropeptide/receptor systems that involve EPACs in different cancers. The EPACs function in different cancer cell lines and models are summarized in Table 1 while the functional roles of neuropeptides are shown in Table 2.

### 3.1. Breast Cancer

Neuropeptide Y (NPY) acts as cancer suppressors in breast cancer, and the effects are mediated by neuropeptide Y receptors 1 and 5 (Y1R and Y5R). Y1R is predominately expressed in breast cancer. NPY/Y1R system was also identified to inhibit forskolin-stimulated cAMP production and mobilize intracellular Ca^2+^ in MCF-7 cells, thus resulting in suppression of oestrogen-induced cancer cell proliferation [84,85].

Y5R expression was observed in the MCF-7, T47D, MDA-MB-231, MDA-MB-468, HS578T, and BT-549 cell lines. In BT-549 cells, activation of Y5R inhibits cAMP production to increase mitogen-activated protein kinase (MAPK) activity with a concomitant increase in extracellular signal-regulated kinase (ERK1/2) phosphorylation. This in turn promotes cell growth [86]. The Y5R blocker, CGP71683A, was indicated to reverse those effects of NPY in the cAMP pathway and induce cell death in BT-549 cells. In murine mammary carcinoma 4T1 cells, NPY was shown to promote a concentration-dependent increase in proliferation through increased phosphorylation of ERK1/2 [88]. The Y5R antagonist L-152,804 was shown to inhibit the proliferation effect of NPY in 4T1 cells [88] while the Y5R-selective antagonist CGP71683A was reported to inhibit the stimulation of MDA MB-231 cell migration [86]. NPY is also involved in pro-angiogenesis by impacting vascular smooth muscle and endothelium [87].

Peptide Y (PYY) was shown to inhibit breast cancer growth through activation of neuropeptide Y receptor 4 (Y4R) [89]. PYY reduces the intracellular cAMP level and suppresses cancer cell proliferation in MCF-7 cells. In turn, there was tumour formation/growth in female athymic NU/NU-*nu*BR mice inoculated with MCF-7 cells [90,91].

These results collectively show that Y1R and Y4R activation inhibits breast cancer cell proliferation through suppression of cAMP accumulation, while Y5R activation stimulates breast cancer cell proliferation through cAMP/MAPK pathway.

VIP was shown to be involved in the promotion of cancer progression. VIP-induced cyto-protection and Bcl2-antagonist of cell death (BAD) phosphorylation were mediated by protein kinase A (PKA) pathways, thus resulting in tumour growth and activation of antiapoptotic signalling in cancer stem cells from breast cancer MCF-7 cells [92].

Gonadotropin-releasing hormone (GnRH) and its receptor (GnRHR) were reported to not only regulate the reproduction process but also modulate cell proliferation and migration in both endocrine and non-endocrine cancers. GnRHR can couple to various Gα subunits such as Gαq/11, Gαi, and Gαs subunits, thus inducing different intracellular secondary messengers and pathways. GnRH was shown to play a dual role in regulating cAMP production in cancer cells. GnRH up-regulates the intracellular concentration of cAMP by Gαs proteins and subsequently activates PKA in gonadotrophic cancers such as ovarian, endometrium, and breast cancer [93]. Interestingly, a high concentration (100 nM) of GnRH agonist inhibits cAMP production right after the initial increase through GnRH receptor [94]. GnRH mediates anti-proliferative effects in breast and prostate cancers [95]. GnRHR activation could induce apoptosis and G2/M arrest and played an anti-proliferative role in breast cancer. These cell cycle, apoptotic, and cytoskeletal-related functions are proposed to be the result of a coordinated dynamic pattern of MAPK signalling [96]. It was also reported that GnRH/GnRHR system suppressed the invasiveness of the highly invasive breast cancer cell line MDA-MB-231 [96].

The expression level of EPAC1 is associated with breast cancer patient prognosis [68]. The protein level of EPAC1 in breast cancer tissues was significantly upregulated versus para-carcinoma tissues. The respective positive rate was 58% versus 10% (*p* < 0.05) [68]. EPAC activator is proved to minimally inhibit MDA-MB231 cell proliferation [67]. In addition, the use of the EPAC antagonist ESI-09 inhibited both EPAC1 and EPAC2, thus leading to cell cycle arrest, apoptosis, and disruption of tubulin distribution via delocalization of A-kinase anchoring protein 9 (AKAP9). This in turn led to suppression of breast cancer cell migration and induction of cell death [69].

To date, there has been no direct evidence for the role of the cAMP/EPAC pathway in breast cancer progression. The mechanism underlying EPAC1/2-induced cell metastatic inhibition remains unclear. Several neuropeptides were found to be involved in either suppression or stimulation of breast cancer progression. The suppression of EPAC1/2 led to inhibition of breast cancer cell migration and stimulation of cell death. These observations suggest that reduction in cAMP levels mediated by neuropeptide/GPCRs system may be related to the suppression of EPAC1/2. This in turn leads to inhibition of breast cancer progression and can potentially correlate to the MAPK and ERK pathways.

### 3.2. Endometrial Adenocarcinoma

Oxytocin (OT) was found to act as an antiproliferation regulator by activating the oxytocin receptor (OTR)/cAMP/PKA pathway in endometrial adenocarcinoma cells. The presence of PKI (6–22) amide—an inhibitor of PKA—suppressed the antiproliferative effect of OT in COLO 684 cells, thus suggesting that the PKA pathway could be the intracellular mediator of the OT effect [97,98]. EPACs are reported to regulate ERK1/2 and calreticulin pathways in human endometrial cells [122,123]. However, research into EPACs function in endometrial adenocarcinoma remains limited, and the role of neuropeptide/GPCR/EPAC axis in endometrial adenocarcinoma remains largely unknown.

### 3.3. Glioblastoma

Vasoactive intestinal peptide (VIP) and pituitary adenylate cyclase activating peptide (PACAP) inactivated the AKT signalling pathway and acted as an anti-invasive factor via pituitary adenylate cyclase activating peptide receptor 1 (PAC1), vasoactive intestinal peptide receptor 1 (VPAC1), and vasoactive intestinal peptide receptor 2 (VPAC2) in the malignant glioblastoma M059K cell line. Of these, cAMP-activated PKA, Rac1, and Cdc42 were suggested to be potential upstream modulators of the phosphorylation of AKT in glioblastoma cells [99]. EPAC1 was reported to play a critical role in glioblastoma progression. This induces cell death and is involved in cell cycle modulation [64]. The cell death of human glioblastoma A172 cells was increased after treatment of 8-pCPT-2′-O-Me-cAMP; cell cycle was arrested at the G2/M phase. In addition, phosphodiesterase inhibitors also functioned via cAMP/EPAC1/Rap1 pathway and regulated A172 cell death and cell cycle [64]. Conversely, EPAC1 was reported to promote glioblastoma proliferation through cAMP/Ras/p44/42 MAPK signalling pathway in U172 and U87MG cells. Furthermore, the inhibition effect of 8-pCPT-2′-O-Me-cAMP in the phosphorylation of p44/42 MAPK was identified to be dose-dependent [62].

In summary, there are few studies into the role of EPAC1/2 in the transduction pathway induced by the neuropeptide/GPCRs system in glioblastoma. However, the AKT signal is mainly regulated by Rap1-mediated signal pathway, and thus inactivation of the AKT signalling pathway mediated by activated neuropeptide/GPCR system may be related to the cAMP/EPAC1/Rap1 pathways in glioblastoma suppression.

### 3.4. Leukaemia

The American Cancer Society and the National Cancer Institute classify leukaemia based on the origin of cell types (lymphoid and myeloid) and tumour development rate (acute and chronic). Consequently, leukaemia is generally classified into four subgroups: acute lymphoblastic leukaemia (ALL), chronic lymphocytic leukaemia (CLL), acute myelogenous leukaemia (AML), and chronic myelogenous leukaemia (CML) [124].

VIP and PACAP suppressed tumour cell proliferation of human leukaemia myeloid cells. VIP and PACAP stimulate AC, phospholipase C (PLC), as well as Ca^2+^ levels in the blasts isolated from patients with myeloid leukaemia through downstream pathways mediated by three receptors: procaspase activating compound 1 (PAC1), VPAC1, and VPAC2 [125]. Exposure of VIP and PACAP increased cAMP level in three out five tested human leukaemia cell lines. The up-regulation of cAMP level was positively co-regulated with *c-fos* and *c-jun* protein expression together with the formation of functional AP-1 transcriptional factor complex [100]. In addition, up-regulation of intra-cytoplasmic calcium levels mediated by both neuropeptides is observed in the human erythroleukemia (HEL) cell line as well as in clinical myeloid leukaemia samples.

Octreotide, a somatostatin (SST) analogue, was reported to inhibit the proliferation of Jurkat cells, a human acute T-cell leukemic cell line, by stimulating cAMP accumulation. A stable somatostatin mitogen, SMS 201995, significantly enhanced the AC activity whereas SRIF28—a SST endogenous SST analogue having almost identical affinity as SMS 201995 for somatostatin SST receptor 3 (SSTR3) —has a contradictory effect [101].

In recent studies, EPAC1 was reported to be involved in the proliferation and apoptosis of leukemic cells: EPAC1 expressed in human B-cell CLL (B-CLL) and human B-cell ALL (B-ALL) as well as the EPAC1 agonist reduced B-CLL apoptosis via the EPAC1/Rap1 pathway—this suggests that EPAC1 signalling played an anti-apoptotic role and enhanced cell survival in B-CLL and B-ALL [71]. In addition, the cAMP/EPAC1 pathway also suppressed B lymphoma cell proliferation via B-cell-receptor-mediated growth arrest and apoptosis in mouse WEHI-231 immature B lymphoma cells. The EPAC1 agonist, 8-pCPT-2′-O-Me-cAMP, reduced DNA synthesis and enhanced the number of apoptotic cells. EPAC1 determined the final cellular apoptotic response by activating Rap1 and H-Ras and participating in the balancing between ERK1/2 and AKT. This maintained the balance between pro-apoptotic and anti-apoptotic signal transduction [70].

The above-mentioned neuropeptides and their receptors could thus modulate the apoptosis of ALL and suppress cell proliferation. However, it remains unclear whether the suppression of cancer cell proliferation by the up-regulation of cAMP levels is dependent on activation of EPAC1, EPAC2, or another intracellular cAMP sensor PKA as well as their downstream pathways.

### 3.5. Lung Cancer

#### 3.5.1. Small Cell Lung Cancer (SCLC)

VIP induces intracellular cAMP level elevation and increases the secretion rate of bombesin-like peptides via VPAC1. This process then stimulates cell proliferation of SCLC in both in vitro and in vivo conditions. Interestingly, the effect of VIP may depend on ligand concentration. Lung cancer cell proliferation was stimulated at low VIP doses (10 nM) while cancer growth was suppressed by high VIP doses (100 nM and 1 μM). This suggests that a high dose of VIP may induce high levels of cAMP, thus leading to increased cell differentiation and a slower cell growth rate [102]. However, the current study into EPACs in SCLC is still missing; thus, the role of EPAC in SCLC remains largely unknown.

#### 3.5.2. Non-Small Cell Lung Cancer (NSCLC)

Similar to SCLC, we found that NSCLC cell proliferation was stimulated by the low dose of VIP (10 nM); cancer growth was reduced by the high concentration of VIP. NSCLC cell proliferation could also be stimulated by neurotensin (NTS) via up-regulation of tyrosine phosphorylation of focal adhesion kinase (FAK) [102]. EPAC1 was shown to promote the degradation of the p300 protein by inhibiting p38 activity in H1299 and A549 lung cancer cells [49]. Association of EPAC1 and β-catenin in the nucleus required direct interaction with the scaffold protein Ezrin during epithelial-to-mesenchymal transition (EMT) [46]. Activation of EPAC1 with 8-pCPT-2′-O-Me-cAMP increased ubiquitin/proteasome-dependent degradation of XRCC1 protein and decreased X-ray repair cross complementing 1 (XRCC1) expression; this led to DNA damage repair. These effects could be attenuated by a knockdown of EPAC1 in lung cancer cells [59]. RNA-binding motif protein 10 (RBM10) inhibits cell proliferation in lung adenocarcinoma by suppressing EPAC/Rap1/AKT/CREB signalling [45]. Isoproterenol-increased HDAC6 expression is also dependent on the PKA and EPAC/ERK pathway and resulted in lung cancer cell migration [47].

### 3.6. Neuroblastoma

Specific OTRs were present in human neuroblastomas and glioblastomas. OT inhibits neuroblastoma and glioblastoma cell proliferation in a dose-dependent manner and were accompanied by a significant up-regulation in intracellular concentration of cAMP [103]. Both EPAC1 and EPAC2 are uniformly distributed in the cell soma and neurites. In contrast to cAMP function, recent evidence suggests that EPACs are mainly involved in neuronal differentiation. Knock-down of EPACs influenced neurite outgrowth in primary rat dorsal root ganglia neurons [126]. The activation of EPACs also contributed to the differentiation of N1E-115 mouse neuroblastoma cells. In N1E-115 differentiating cells, spatial and temporal regulation of EPAC1 and 2/Rap1 signalling was required to promote neurite outgrowth and extension [127]. EPAC2 functioned via EPAC2/Ras signalling and reduced basal dendritic architecture in cortical neurons [128]. Furthermore, EPAC2 was regarded as a more critical factor than EPAC1 in promoting neurite elongation [127].

### 3.7. Ovarian Cancer

In human ovarian tissue, a high expression of the NPY receptors Y1R and neuropeptide Y receptor 2 (Y2R) was identified in 10 inhibin-expressing granulosa cell tumours, Leydig cell tumours, and Sertoli-Leydig cell tumours. NPY-activated Y1R and Y2R might play a role in the pathogenesis and pathophysiology of ovarian malignancies [104]. NPY were shown to down-regulate intracellular cAMP levels by inhibiting AC activity [129]. Although the relationship between the neuropeptide/GPCR system and EPAC remains unknown, the NPY-induced down-regulation of cAMP level might be related to inhibition of ovarian cancer cell proliferation.

EPAC1 knockdown was shown to inhibit the proliferation of ovarian cancer cells such as SKOV3 and OVCAR3. This inhibition might be mediated by EPAC1-knockdown-induced G1 phase arrest by suppressing the AKT/Cyclin D1/CDK4 pathway [73]. EPAC1-regulated cancer cell migration was also seen in a Rap1-dependent manner in ES-2 and OVCAR3 cell lines [74,75,76]. In addition, epithelial wound migration and carcinoma invasion was shown to be modulating by the cAMP/EPAC/Rap1 pathway upon regulation of laminin-5 and α3β1 integrins [76].

### 3.8. Prostate Cancer

The NPY/Y1R system inhibited cancer cell proliferation in LNCaP and DU145 prostate cancer cell lines, but the opposite effect was found in PC3 cells. The signalling pathways of NPY are also varied in these cell lines. NPY treatment had no effect on elevated constitutive levels of phosphorylated ERK1/2 in LNCaP cells. However, there was long-lasting ERK1/2 activation stimulated by NPY in DU145 cells. In contrast, a rapid and transient ERK1/2 that required activation of protein kinase C (PKC) was observed in PC3 cells. In addition, reduction of forskolin-stimulated cAMP accumulation induced by NPY treatment was only identified in PC3 cells. Intracellular Ca^2+^ concentration was not affected by NPY treatment in these three prostate cancer cell lines [106]. In another study, the NPY-activated signal was involved in robust proliferation-independent transcriptional changes and ERG rearrangements in prostate cancer [107].

VIP was reported to induce *c-fos* expression by enhancing intracellular Ca^2+^ levels, thus resulting in the expression of VEGF in LNCaP cells. VIP-induced effects were completely blocked by the treatment of PKA inhibitor (H89) together with the intracellular calcium chelator BAPTA/AM; each agent alone led only to a partial inhibition [108]. Furthermore, activation of VPAC1 by VIP stimulates the expression of angiogenic VEGF, the pro-inflammatory enzyme COX-2, and the increased activity of MMP-2 and 9 in tumours derived from VIP-treated PC3 cells [109].

In LNCaP cells, calcitonin (CT) induces a dose-dependent up-regulation of intracellular cAMP with a rapid and sharp increase in cytoplasmic Ca^2+^ concentrations. This led to cancer cell proliferation. A locally secreted CT-like peptide(s) also induced mitogenic responses in prostate cancer cells, thus leading to the accumulation of cAMP and calcium [110,111]. Exogenous CT treatment increased the in vitro invasion of PC3 cells mediated by a rapid, large (several-fold), but transient increase in PKA activity. Importantly, CT increased the invasiveness of LNCaP and PC-3M-CT cells but did not affect PC-3 and PC-3M-Gsα cells. These results suggest that the action of CT might be mediated by PKA signalling, which subsequently increased cell invasion and secretion of gelatinases [112].

Adrenomedullin (AM) was reported to stimulate cell proliferation in vitro through the cAMP/CRAF/MEK/ERK pathway. Anti-AM antibody (αAM) treatment suppressed DU145 and PC-3 cell proliferation, suggesting that AM might function as a potent autocrine/paracrine growth factor in prostate cancer androgen-independent cells. The growth of DU145 androgen-independent xenografts and castrated LNCaP androgen-dependent xenografts were suppressed by αAM treatment, suggesting that AM might have a strong effect in tumour regrowth following androgen ablation [113,114].

Activation of the GnRH/GnRHR axis decreases cell proliferation in prostate cancer in a local paracrine/autocrine manner [115,116,117]. In LNCaP and DU145 cells, the signalling pathway activated by the GnRH/GnRHR axis inhibits cell proliferation and crosstalk with epidermal growth factor receptor (EGFR) and insulin-like growth factor 1 (IGF-1) [118]. GnRHR was previously reported to link to the Gαi/cAMP signal transduction pathway: GnRH-I or its antagonist could inhibit FSK-mediated cAMP accumulation in BPH-1 cells [119,120].

In prostate cancer, EPAC1 was found to play a complex role in cancer cell proliferation, migration, and angiogenesis via different pathways. The 8-pCPT-2′-O-Me-cAMP increase mTORC2-dependent AKT phosphorylation at S473 in a dose-dependent manner and mediated cell death and cell survival [77]. What’s more, EPAC1 was shown to function as a “scaffold” protein. It formed a multiprotein signalling complex with AKAP, Raptor, PDE3B, PDE4D, and Rictor through an immunoprecipitation assay [77]. Up-regulation of cell proliferation and survival was stimulated by the EPAC1 agonist, 8-pCPT-2′-O-Me-cAMP, via activation of Ras-MAPK and PI3K/AKT/mTOR signalling cascades [83] EPAC1-regulated COX-2-dependent pathways formed a pro-proliferative feedback loop together with MAPK and cPLA2 pathways [83]. Furthermore, the EPAC/Rap1/B-Raf signalling pathway was reported to be the AC-dependent signalling pathway and regulates the proliferation of prostate carcinoma cells [80].

In contrast, cancer cell proliferation and migration was inhibited by down-regulating the MAP kinase and RhoA signalling pathways after 8-pCPT-2′-O-Me-cAMP treatment [78]. However, partial depletion of EPAC1 and EPAC2 by siRNAs had no effect on the secretion of VEGF with only minor inhibitory effects on tumour growth in PC3-cRap1 cells. Although the 8-pCPT-2′-O-Me-cAMP affinity for EPAC1 was 107-fold greater than that of PKA, the suppression of PKA activity could reverse the inhibition of the tumour growth and angiogenesis mediated by 8-pCPT-2′-O-Me-cAMP *in vivo*. These results suggest that the inhibition effect in tumour growth might be caused by PKA activated by 8-pCPT-2′-O-Me-cAMP [130]. In addition, both cAMP effectors, EPAC and PKA, were involved in different NF-κB1-VIP pathways corresponding to the tumour or non-tumour situation in prostate epithelial cells. VIP can function via cAMP/PKA in non-tumour cells as well as cAMP/EPAC/ERK/PI3K in tumour cells [131].

NPY has induced diverse signalling pathways and effects in different prostate cancer cell lines. The inhibition of cancer cell proliferation has been regulated by NPY-induced long-lasting ERK1/2 activation. NPY-induced rapid and transient ERK1/2 activation was seen upon regulating the stimulation of cancer cell proliferation. The contrast effect of NPY on prostate cancer cell proliferation remains unclear. AM increased cell proliferation and invasiveness via cAMP and CRAF/MEK/ERK pathways. Other than neuropeptide-induced cAMP-mediated tumour progression, only the mechanism underlying VIP-induced tumour growth has been well studied and mediated via cAMP/EPAC/ERK/PI3K pathways in tumour cells.

### 3.9. Renal Cancer

Activation of arginine vasopressin receptor 2 (V2R) by arginine vasopressin (AVP) stimulates the cell proliferation of renal cell carcinoma (RCC). The V2R antagonists Tolvaptan and OPC31260 can silence V2R and have been shown to reduce wound closure and cell viability of the 786-O and Caki-1 human clear cell RCC (ccRCC) cell lines. In addition, Tolvaptan and OPC31260 treatment decreased RCC tumour growth and reduced angiogenesis in vitro. This in turn increased apoptosis in xenograft models. In contrast, the V2R agonist dDAVP significantly increases tumour growth in Caki-1-implanted xenograft models. High levels of intracellular cAMP and ERK1/2 activation were observed in human clear cell RCC tumours. V2R agonists reduce cAMP and ERK1/2 activation in mouse tumours and Caki-1 cells, while dDAVP treatment had the opposite effect. V2R gene silencing in Caki-1 cells also reduced cAMP and ERK1/2 activation [121]. EPAC1 is highly expressed, and was highly correlated with PDE4, AKAP95, CX43, cyclin E1, and cyclin D1 in the rectal carcinoma tissues versus normal tissues [61]. In addition, EPAC1 was reported to inhibit cell proliferation in the ccRCC A498 cells [67]. In summary, the cAMP/ERK1/2 pathway triggered by V2R activation stimulates cell proliferation in RCC cell lines.

## 4. Discussion

It is widely recognized that the neuropeptide/GPCR family members act as tumour regulators for multiple types of cancer. Studies on the role of the neuropeptide/GPCR system in cancer development underscore the complexity of the neuroendocrine/GPCR/cAMP pathway. Multiple neuropeptide GPCRs are essential intracellular cAMP level modulators; thus, it is necessary to investigate the relationship between those neuropeptide/GPCR-systems as well as the down-stream pathways mediated by the cAMP effectors EPAC and PKA. We summarized the reported neuropeptide/GPCR systems that trigger cAMP pathway in cancer cells. Several neuropeptides are well-known in modulating cancer cell signalling. For example, VIP is reported to function via cAMP/PKA in non-tumour cells, and via cAMP/ERK/PI3K in prostate tumour cells [131]. Moreover, reports also demonstrated the function of neuropeptides in nervous system could be modulated by EPACs pathway, such as NPY is reported to regulate fear behaviours, human depression, and other neuropsychiatric disorders via EPAC pathways [132,133,134,135]. However, only a few studies have identified the neuropeptide/EPAC1/2 axis in limited types of cancer. In addition, our understanding about EPAC pathways is mainly focused on the EPAC1 Evidence about the function of EPAC2 in cancer cells are largely unknown. Since the EPAC1 and EPAC2 may perform differently and leading to diverse functional impact to cancer cells, systematic analysis of EPAC1/2, as well as PKA after the activation of cAMP production will be one of the important directions in cancer research. In conclusion, EPAC is a major effector of cAMP that could be induced by neuropeptide/GPCR systems in cancer cells even though there are few studies investigating this connection. Continued research should clarify the modulation of EPAC via neuropeptide-induced cAMP regulation.

## Figures and Tables

**Table 1:** EPACs functions in tumour development (↑ refers to up-regulation, ↓ refers to down-regulation).

Cancer Type	Cell Line/Model (s)	Downstream Pathway (s)	Function (s)	Reference (s)
Lung cancer	A549 and H1299	EPAC1/Rap1/AKT/CREB	↑ Cell proliferation	[45]
	A549	EPAC1/Rap1/Rac1/β-catenin,	↑ Migration/Metastasis	[46,47]
		EPAC1/Rap1/Raf/MEK/ERK/HDAC6		
	A549	EPAC1/XRCC1	↑ XRCC1 degradation ↑ DNA damage repair	[48]
	A549 and H1299	EPAC1/P38/MAPK/P300	↑ Promote P300 protein degradation	[49]
Melanoma	A375 and MeWo	EPAC1/RP1/ERK/αvβ3 integrin	↑ Migration/Metastasis	[50]
	SK-Mel-2 and SK-Mel-24	EPAC1/Heparan Sulfate	↑ Migration/Metastasis	[51,52]
	SK-Mel-24	EPAC1/Ca^2+^	↑ Cell proliferation	[53,54]
Pancreatic cancer	PANC-1	EPAC1/2	↓ Cell proliferation ↑Apoptosis	[55]
	PANC-1	EPAC1/Cell ruffling/Paxillin focal adhesions, Integrin β1	↑ Migration/Metastasis	[41,56,57]
Cervical cancer	Hela	EPAC1/Rap1/Rac1	↑ Migration/Metastasis ↑ Lamellipodia formation	[58]
Fibrosarcoma	HT1080	EPAC1/Rap1/Rac1/ATX/LPA4	↑ Migration/Metastasis	[59]
Gastric cancer	Patients’ tissue, SK-GT-2, GC	N.A.	↑ Cell proliferation	[60]
Rectal cancer	Patients’ tissue	PDE4/cAMP/EPAC1	↑ Cell proliferation	[61]
Glioblastoma	A172 and U87MG	EPAC1/Rap1, MAPK	↑ Cell death ↑ Cell cycle arrest	[62,63,64]
Bladder cancer	UMUC3, tumour tissues	EPAC1/Rap1	↓ Cell migration	[65]
Renal cancer	A498	EPAC1/PI3K	↓ Cell proliferation	[66]
Breast Cancer	MDA-MB-231	N.A.	↓ Cell proliferation	[67,68]
	MCF-7	EPAC1 and EPAC2 activates AKAP9	↑ Cell adhesion ↑ Migration/Metastasis	[69]
Leukemia	WEHI-231	EPAC1/Rap1/H-Ras/ERK1/2	↓ Cell proliferation	[70]
	B-CLL and ALL	EPAC1/Rap1	↑ Cell proliferation ↓ Apoptosis	[71,82]
Neuroendocrine cancer	QGP-1	EPAC1/Rap1/Cyclin D1 and p27	↑ Cell proliferation	[72]
	H727	EPAC1/Rap1/Cyclin D1 and P27	↓ Cell proliferation	[72]
	QGP-1 and H727	EPAC1/Rap1	↑ Cell adhesion	[72]
Ovarian cancer	SKOV3 and OVCAR3	EPAC1/PI3K/AKT/Cyclin D1/CDK4	↑ Cell proliferation	[73]
	ES-2	EPAC1/Rap1/CERB	↓ Migration/Metastasis	[74]
	OVCAR3	EPAC1/Rap1/Integrins	↑ Migration/Metastasis	[75,76]
Prostate cancer	PC-3 and DU 145	EPAC1/MAPK	↓ Cell proliferation ↓ DNA synthesis	[78]
	1-LN, DU-145 and PC-3	EPAC1/ERK/AKT/mTOR	↑ Cell proliferation	[77,79,83]
	LNCaP and PC3	EPAC1/Rap1/B-Raf Cyclin B1 and CDK1	↑ Cell proliferation	[80]

**Table 2:** Neuropeptides involving in tumour development through cAMP related pathways (↑ refers to up-regulation, ↓ refers to down-regulation).

Cancer Type	Neuropeptides	Receptor (s)	Cell Line/Model (s)	Downstream Pathway (s)	Function (s)	Reference (s)
Breast cancer	NPY	Y1R	MCT-7, 4T1	−cAMP	↓ Cell proliferation	[84,85]
		Y5R	BT-549, 4T1, primary breast neoplasia tissue	+MAPK and ERK1/2	↑ Cell proliferation ↑ Metastasis	[86,87,88]
	PYY	Y4R	MCT-7	−cAMP	↓ Cell proliferation	[89,90,91]
	VIP	VPAC1, VPAC2	Cancer stem cells from MCT-7	+cAMP/PKA	↑ Tumour growth ↓ Apoptosis	[92]
	GnRH	GnRHR	MCT-7, MDA-MB-231	+cAMP/PKA	↓ Cell proliferation ↑ Apoptosis ↓ Invasiveness	[93,94,95,96]
Endometrial Adenocarcinoma	OT	OTR	Colo 684, A-MEC, HEC1A, and Ishikawa cells, human endometrial neoplasm tissues	+cAMP/PKA	↓ Cell proliferation	[97,98]
Glioblastoma (GBM)	VIP/PACAP	PAC1 receptor, VPAC1 and VPAC2	M059J, M059K, U87	+AKT (in M059K cells) +AKT via PKA, Rac1, Cdc42	↓ Invasiveness	[99]
Leukemia	VIP	VPAC1 and VPAC2	human erythroleukaemia (HEL)	+AC and PLC	↓ Cell proliferation	[100]
	PACAP	PAC1, VPAC1 and VPAC2	human myeloid leukaemia	+Ca^2+^ level	↓ Cell proliferation	
	SST analog (Octreotide)	SSTR	Jurkat cells	+AC	↓ Cell proliferation	[101]
Small cell lung cancer (SCLC)	VIP	VPAC1	SCLC, SCLC patients samples	+AC	↑ Cell proliferation (Low doses)	[102]
					↓ Cell proliferation (High doses)	
Non-small-cell lung cancer (NSCLC)	VIP	VPAC1	NSCLC, NSCLC patient samples	+AC	↑ Cell proliferation (Low doses)	[102]
					↓ Cell proliferation (High doses)	
Neuroblastoma	OT	OTR	SK-N-SH, SH-SY5Y, IMR-32 and MOG-G-UVW	+cAMP	↓ Cell proliferation	[103]
Ovarian cancer	NPY	Y1R, Y2R	Human ovarian tissue	−AC −cAMP levels	↑ Cell proliferation ↑ Angiogensis	[104,105]
Prostate cancer	NPY	Y1R	PCa cell lines (LNCaP, DU145, PC3)	+ERK1/2 (in DU145 cells) −cAMP, + ERK1/2 via PKC (in PC3 cells)	↓ Cell proliferation (in LNCaP, DU145 cells) ↑ Cell proliferation (in PC3 cells)	[106,107]
	VIP	VPAC1	LNCaP, PC3, PC3 nude mice xenograft	+PKA	↑ Tumour growth	[108,109]
	CT	CTR	PC-3M, LNCaP and PC-3	+cAMP and Ca^2+^	↑ Cell proliferation ↑ Invasiveness	[110,111,112]
	AM	AMR, CLR, RAMP2 and RAMP3	LNCaP, DU145 and PC-3, xenografts model	+cAMP and CRAF/MEK/ERK	↑ Cell proliferation ↑ Invasiveness	[113,114]
	GnRH	GnRHR	LNCaP, DU145, and BPH-1	−cAMP	↓ Cell proliferation	[115,116,117,118,119,120]
Renal cell carcinoma (RCC)	AVP	VR	786-O and Caki-1 cells, mouse xenografts	+cAMP +ERK1/2	↑ Cell proliferation	[121]

## Data Availability

Not applicable.
